# Aging Influences Hepatic Microvascular Biology and Liver Fibrosis in Advanced Chronic Liver Disease

**DOI:** 10.14336/AD.2019.0127

**Published:** 2019-08-01

**Authors:** Raquel Maeso-Díaz, Martí Ortega-Ribera, Erica Lafoz, Juan José Lozano, Anna Baiges, Rubén Francés, Agustín Albillos, Carmen Peralta, Juan Carlos García-Pagán, Jaime Bosch, Victoria C Cogger, Jordi Gracia-Sancho

**Affiliations:** ^1^Liver Vascular Biology Research Group, Barcelona Hepatic Hemodynamic Laboratory, IDIBAPS Biomedical Research Institute, University of Barcelona Medical School, Barcelona, Spain; ^2^Biomedical Research Network Center in Hepatic and Digestive Diseases (CIBEREHD), Madrid, Spain; ^3^Instituto de Investigación Sanitaria y Biomédica de Alicante (ISABIAL -Fundación FISABIO), Alicante, Spain; ^4^ Department of Gastroenterology and Hepatology, Hospital Universitario Ramón y Cajal, IRYCIS, Universidad de Alcalá, Madrid, Spain; ^5^Protective Strategies Against Hepatic Ischemia-Reperfusion Group, IDIBAPS, Barcelona, Spain; ^6^Hepatology, Department of Biomedical Research, Inselspital, Bern University, Switzerland; ^7^Centre for Education and Research on Ageing & ANZAC Research Institute, University of Sydney and Concord Hospital, Sydney, Australia

**Keywords:** Cirrhosis, portal hypertension, hepatic sinusoid, elderly, liver microcirculation

## Abstract

Advanced chronic liver disease (aCLD) represents a major public health concern. aCLD is more prevalent and severe in the elderly, carrying a higher risk of decompensation. We aimed at understanding how aging may impact on the pathophysiology of aCLD in aged rats and humans and secondly, at evaluating simvastatin as a therapeutic option in aged animals. aCLD was induced in young (1 month) and old (16 months) rats. A subgroup of aCLD-old animals received simvastatin (5 mg/kg) or vehicle (PBS) for 15 days. Hepatic and systemic hemodynamic, liver cells phenotype and hepatic fibrosis were evaluated. Additionally, the gene expression signature of cirrhosis was evaluated in a cohort of young and aged cirrhotic patients. Aged animals developed a more severe form of aCLD. Portal hypertension and liver fibrosis were exacerbated as a consequence of profound deregulations in the phenotype of the main hepatic cells: hepatocytes presented more extensive cell-death and poorer function, LSEC were further capillarized, HSC over-activated and macrophage infiltration was significantly increased. The gene expression signature of cirrhosis significantly differed comparing young and aged patients, indicating alterations in sinusoidal-protective pathways and confirming the pre-clinical observations. Simvastatin administration for 15-day to aged cirrhotic rats improved the hepatic sinusoidal milieu, leading to significant amelioration in portal hypertension. This study provides evidence that aCLD pathobiology is different in aged individuals. As the median age of patients with aCLD is increasing, we propose a real-life pre-clinical model to develop more reliable therapeutic strategies. Simvastatin effects in this model further demonstrate its translational potential.

The socioeconomic and medical care improvement during the last decades has led to a relevant increase in the elderly population around the world. This growth of older population will continue to outpace that of younger population over the next years [1]. Aging is associated with a physiological decline in most organ functions, including the liver [2]. Indeed, we have recently described the changes occurring in the liver sinusoidal cells during healthy aging, demonstrating that aging is accompanied by an accumulation of slight, but significant, modifications in the hepatic sinusoid that could turn into vulnerability in front of chronic or acute liver damage [3]. Advanced chronic liver disease (aCLD) represents a serious and costly problem for our society. Approximately 844 million people suffer from a chronic liver condition worldwide, making it comparable to other major public health problems related to chronic diseases such as cardiovascular diseases [4, 5]. The incidence of aCLD increases dramatically with age, and is accompanied by worse prognosis [6], consequently aging has been defined as a major risk factor for the development of chronic liver conditions [7, 8]. Importantly, and despite the impact of aging on human aCLD, most of the pre-clinical studies aimed at understanding liver disease pathophysiology have been developed in young animals, with few exceptions [9-11].

Statins have been proposed for the treatment of aCLD. Indeed, pre-clinical studies suggested sinusoidal-protective properties [12-16], which may explain the benefits of statins on portal hypertension, survival and hepatocellular carcinoma development observed in clinical and epidemiological studies [17-19]. Nevertheless, all published pre-clinical studies were made in young animals while clinical studies included patients with an advanced median age.

The present study aimed at understanding how aging may impact on the pathobiology of aCLD, specially focusing on the hepatic microcirculatory dysfunction, fibrosis and portal hypertension. We hypothesized that the cumulative mild changes observed in the sinusoidal milieu of healthy aged livers may have relevant consequences during chronic liver injury.

Secondary aims included the evaluation of simvastatin as a therapeutic option in a pre-clinical rat model of aged cirrhosis, and to elucidate the mechanisms responsible for the possible beneficial effect of the treatment.

## MATERIALS AND METHODS

Additional materials and methods are included in the online supplementary information.

### Induction of cirrhosis & simvastatin treatment

Advanced chronic liver disease was induced in male Wistar rats 1-month old (young group; aCLD-young) and 16-month old (aged group; aCLD-old) through chronic exposure to CCl_4_ and phenobarbital [16, 20].

After approximately 14 weeks, once the animals developed ascites, CCl_4_ and phenobarbital administration was discontinued. No differences in the hepatotoxicants administration period to achieve aCLD were observed comparing groups. 5 days after detection of ascites, aCLD-young and aCLD-old animals (n=7 per group) were exhaustively characterized to accomplish the first objective of the study. An additional group of aCLD-old animals (n=20) were distributed randomly to investigate the effects of simvastatin on portal hypertension, microcirculatory dysfunction and fibrosis in the aged cirrhotic model. Simvastatin (5 mg/kg) or its vehicle (PBS) was administered daily by gavage for 15 days. Hemodynamic analysis and subsequent sample processing were performed 1 hour after the last administration. Simvastatin or its vehicle was prepared by a third person, and therefore, the investigators administering the drug and performing the experiments were not aware of the treatment received by the rats. This blinding was maintained until the final analysis of results. Animals were kept in environmentally controlled animal facilities. All procedures were approved by the laboratory animal care and use committee of the University of Barcelona and were conducted in accordance with the European Community guidelines for the protection of animals used for experimental and other scientific purposes (EEC Directive 86/609)

### *In vivo* haemodynamic measurements

Mean arterial pressure (MAP), portal pressure (PP) and portal blood flow (PBF) were measured in old and young rats using micro-catheters and transit-time flow probes [14]. Hepatic vascular resistance (HVR) was calculated as PP/PBF.

### Liver endothelial function

After *in vivo* hemodynamic measurements, livers were quickly isolated and perfused. Liver endothelial function was determined as response to incremental doses of the endothelium-vasodilator acetylcholine [21].

### Hepatic cells isolation

Hepatocytes, Kupffer cells (KC), Liver Sinusoidal Endothelial Cells (LSEC), and Hepatic Stellate Cells (HSC) were isolated using well-established protocols [22]. Only highly pure and viable cells were used.

### Electron microscopy

Liver sinusoidal ultrastructure was characterized using electron microscopy as previously described [3, 23].

### Histological analysis

Liver samples were fixed in 10% formalin, embedded in paraffin, sectioned, and slides were stained with hematoxylin and eosin (H&E) to analyze the hepatic parenchyma, with Sirius Red for liver fibrosis evaluation, with Oil red-O for lipid quantification or with corresponding antibodies for protein immunohistochemistry (IHC) or immunofluorescence (IF).

**Table 1:** Biometric, biochemical and hemodynamic characteristics in aCLD-young and aCLD-old rats.

	aCLD-young 4 m.o.	aCLD-old 20 m.o.	% change	p-value
**Body weight (g)**	371 ± 16	701 ± 35	+88	**<0.001**
**Liver (g)**	10.7 ± 0.6	17.9 ± 0.9	+67	**<0.001**
**Liver-body weight ratio (%)**	2.88 ± 0.13	2.57 ± 0.14	-11	0.09
**AST (U/L)**	189 ± 11	304 ± 47	+61	**0.03**
**ALT (U/L)**	82 ± 8	97 ± 8	+20	0.17
**Bilirubin (mg/dL)**	0.51 ± 0.24	0.51 ± 0.12	0	>0.20
Bile production (µL/min*g)	30 ± 8	18 ± 3	-40	0.16
**Albumin (mg/dL)**	26 ± 1	20 ± 2	-24	**0.01**
**Plasma cholesterol (mg/dL)**	75 ± 5	106 ± 11	+41	**0.01**
**Plasma LDL cholesterol (mg/dL)**	45 ± 4	75 ± 8	+66	**0.007**
**Plasma HDL cholesterol (mg/dL)**	20 ± 2	13 ± 2	-33	**0.03**
**Plasma triglycerides (mg/dL)**	41 ± 8	84 ± 13	+107	**0.01**
**Plasma FFA (mg/dL)**	623 ± 67	504 ± 70	-19	>0.20
**Oil red O-staining (%)**	0.47 ± 0.14	1.83 ± 0.32	+289	**0.04**
**MDA (nmol/mg protein)**	2.68 ± 0.68	2.36 ± 0.28	-12	>0.20
**LPS (EU/mL)**	1.13 ± 0.19	2.00 ± 0.13	+77	**0.002**

**PP (mmHg)**	14.3 ± 0.3	16.9 ± 1.2	+18	**0.03**
PBF (mL/min*g)	1.21 ± 0.12	1.59 ± 0.23	+31	0.12
HVR (mmHg*min/mL*g)	12.7 ± 1.4	12.5 ± 2.3	-2	>0.20
*Ex vivo* HVR (mmHg*min/mL*g)	0.23 ± 0.03	0.41 ± 0.04	+78	**0.002**
**MAP (mmHg)**	91 ± 6	103 ± 7	+13	>0.20
**HR (bpm)**	340 ± 15	338 ± 16	-0.5	>0.20

Data expressed as mean ± S.E.M. (n = 7 each group). AST: aspartate transaminase; ALT: alanine transaminase; LDL: low density lipoprotein; HDL: high density lipoprotein; FFA: free fatty acids; MDA: malondialdehyde; LPS: lipopolysaccharide; EU: endotoxin units; PP: portal pressure; PBF: portal blood flow; HVR: hepatic vascular resistance; MAP: mean arterial pressure; HR: heart rate.

**Figure 1.**
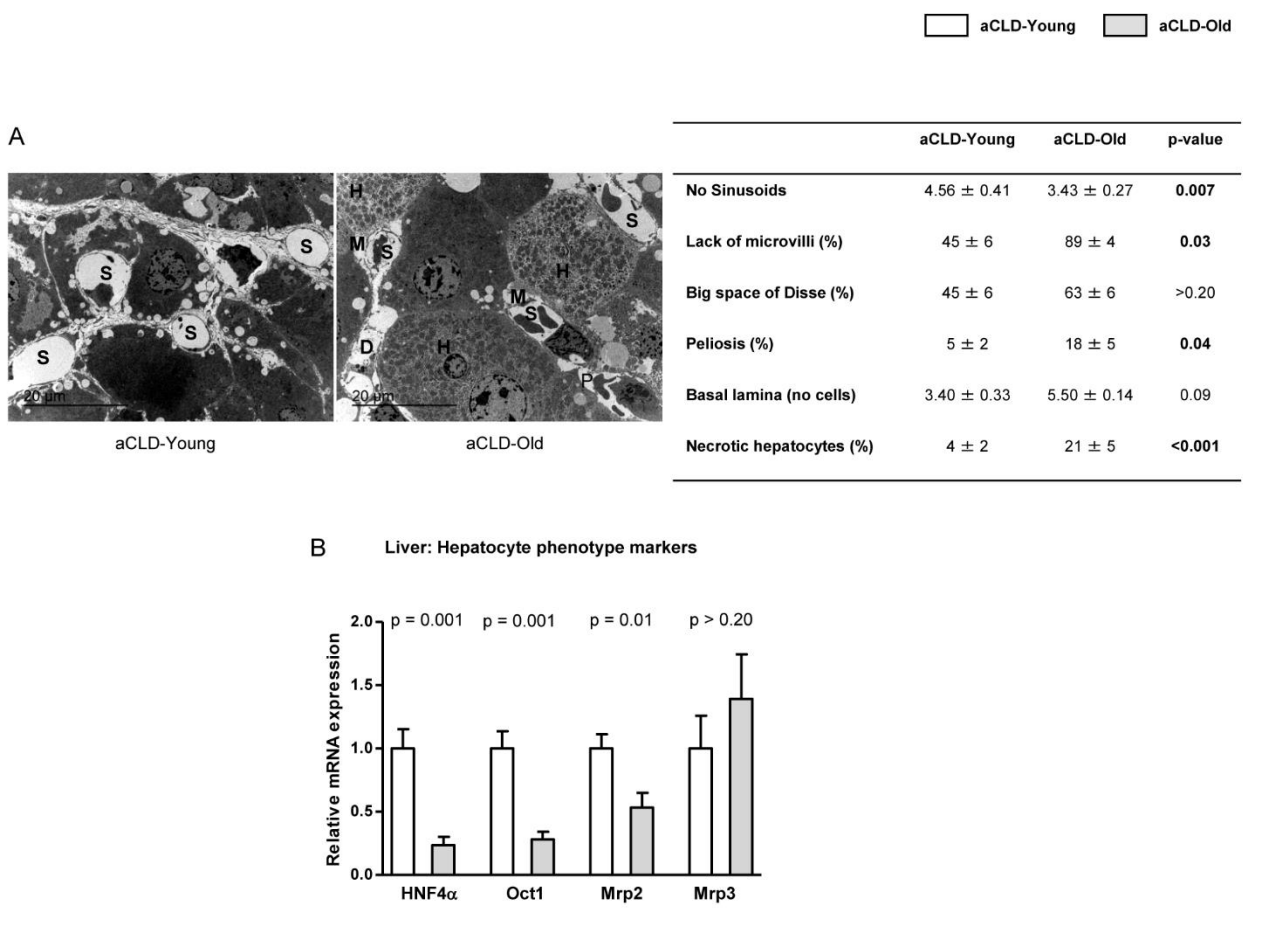
Hepatocyte phenotype markers in 4 months-young and 20 months-old rats with aCLD (**A**) Representative transmission electron microscopy images and corresponding quantification of numbers of sinusoids *(S)*, lack of microvilli *(M)*, big space of Disse *(D)*, peliosis*(P)*, basal lamina deposition and number of necrotic hepatocytes *(H)* in liver tissue from 4 months-young and 20 months-old rats with aCLD. (**B**) HNF4α, Oct1, Mrp2 and Mrp3 mRNA expression in livers described in A. n=3 (A) and (B) n=7 per group. Results represent mean ± S.E.M. All images: 3000X, scale bar=20μm.

### Nitric oxide and superoxide determinations

Levels of cGMP, marker of nitric oxide bioavailability, were analyzed in liver homogenates using an enzyme immunoassay following manufacturer instructions (Cayman Chemical Co., Ann Arbor, MI) [24]. *In situ* mitochondrial or total superoxide levels in hepatic tissue or in cells were assessed with the mitochondrial oxidative fluorescent dye MitoSOX (MitoSOX 5µM; Molecular Probes Inc., Eugene, OR) or with the total oxidative fluorescent dye dihydroethidium (DHE 10µM; Molecular Probes Inc., Eugene, OR) respectively as described [25, 26]. Fluorescence images were obtained with a fluorescence microscope (Olympus BX51, Tokyo, Japan), and quantitative analysis of at least 20 images per condition containing equivalent number of cells was performed with Image J 1.44m software.

### Human liver mRNA analysis

Fractions of liver biopsy specimens obtained by transjugular route, and primarily processed for clinical pathology, were stored in diethyl pyrocarbonate (DEPC) solution for mRNA isolation using the RNeasy kit (Qiagen). 250 ng of highly pure and preserved RNA were deeply analyzed using the Illumina Whole Genome-DASL assay, which quantifies approximately 24,000 transcripts. The microarray data were deposited and stored in GEO (GSE77627).

Illumina were processed using lumi package quantile normalisation [27]. Coefficient for age, derived from a linear model using probe set expression versus age and gender adjusted [28], was employed as a metric score to evaluate the influence of age in the gene expression from cirrhotic liver tissue. We performed pre-ranked gene set enrichment analysis (GSEA) using the canonical pathways MSigDB collection signatures [29].

Ethics Committee of the Barcelona Hospital Clinic approved the experimental protocol (HCB/2011/6814). Experimental groups were defined considering patients’ age: young (n=7, mean age 42 ± 5 years old, range 33-48), old (n=7, mean age 62 ± 4, range 58-70).

### Statistical Analysis

Statistical analysis was performed with the SPSS for Windows statistical package (IBM, Armonk, New York, USA). All results are expressed as mean ± standard error of the mean (S.E.M.). Comparisons between groups were performed with Student’s *t* test. Differences were considered significant at a p value <0.05.

**Figure 2.**
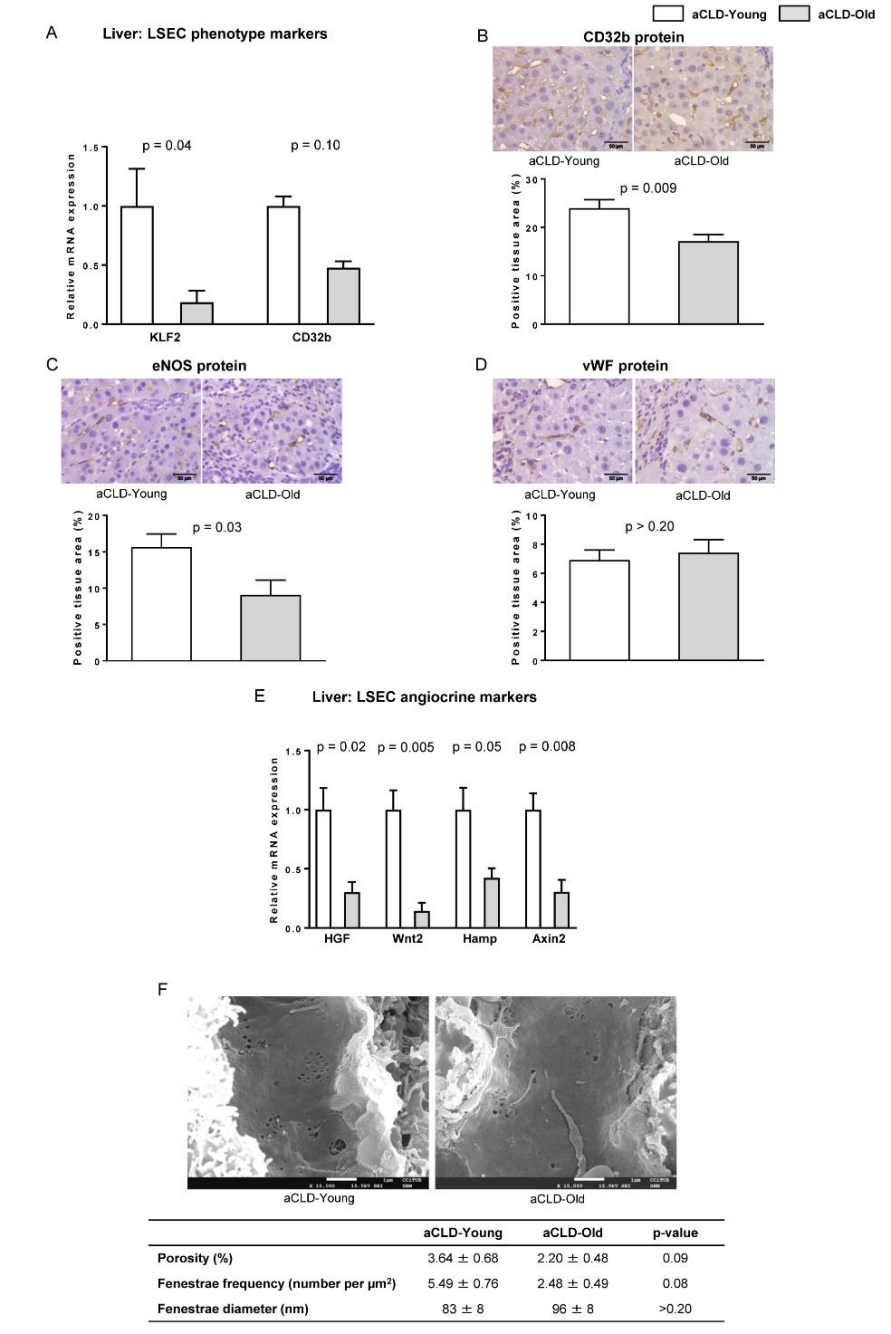
LSEC phenotype markers in aged rats with aCLD The following markers of sinusoidal endothelial phenotype were analysed in liver tissue from 4 months-young and 20 months-aged rats with aCLD. (**A**) mRNA expression of KLF2 and CD32b. (**B**) Representative images of CD32b immunehistochemistry and corresponding quantification. (**C**) Representative images of eNOS immunohistochemistry and corresponding quantification. (**D**) Representative images of vWF immunohistochemistry and corresponding quantification. (**E**) mRNA expression of HGF, Wnt2, Hamp and Axin2. (**F**) Representative scanning electron microscopy images & quantification of porosity, fenestration frequency and fenestration diameter. n=7 (A-E) and n=3 (F) per group. Results represent mean ± S.E.M. Images from B-D: 400X, scale bar=50μm. Images from F: 15000X, scale bar=1μm.

**Figure 3.**
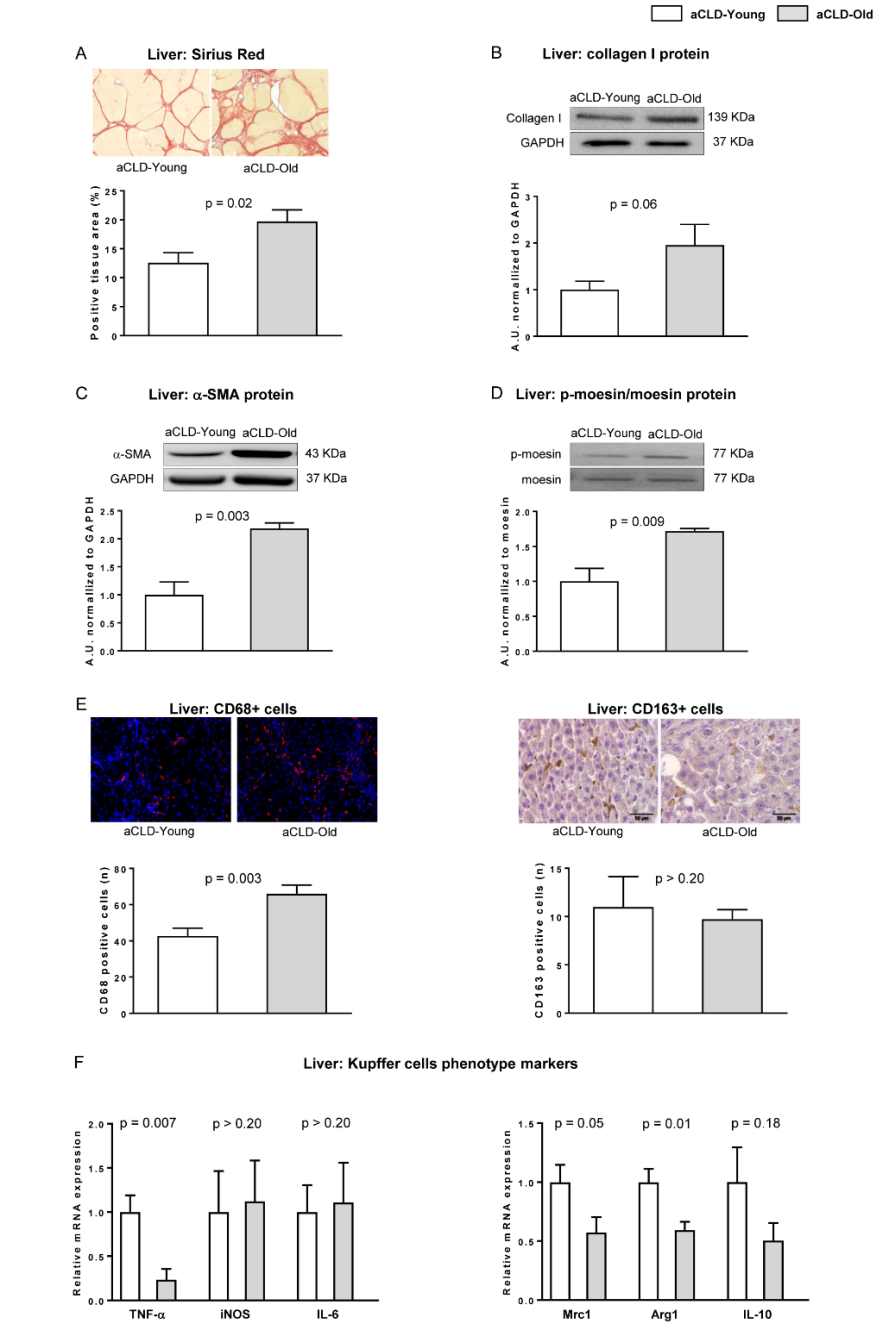
Aging increases fibrotic deposition, HSC activation and macrophages infiltration Fibrotic content, HSC phenotype and macrophage infiltration and phenotype were evaluated in young and aged rats with aCLD. (**A**) Representative images of fibrotic content measured as positive area for Sirius Red with their corresponding quantifications. (**B**) Representative western blot of Collagen I normalized to GAPDH. (**C**) Representative western blot of α-SMA normalized to GAPDH. (**D**) Representative western blots of moesin and p-moesin and corresponding quantification. (**E**) *Left*, representative images of CD68 immunofluorescence in liver tissue and corresponding quantification. *Right*, representative images of CD163 immunohistochemistry in liver tissue and its quantification. (**F**) Expression of TNF-α, iNOS, and IL-6 as pro-inflammatory markers (left) and Mrc1, Arg1 and IL-10 as anti-inflammatory markers (right) in liver tissue from young and old rats with aCLD. n=7 per group. Results represent mean ± S.E.M. All images: 400X, scale bar=50μm.

## RESULTS

### Aged rats with aCLD: baseline biometric and biochemical characteristics

As shown in Table 1, old rats with aCLD showed an increase in body and liver weight compared to young aCLD animals; however, liver/body-weight ratio was moderately diminished. Evaluation of hepatic function suggested a more severe deterioration in the aging group, shown by lower plasma albumin levels and bile production and higher serum transaminases.

Evaluation of plasma lipids revealed higher total cholesterol, LDL-cholesterol, HDL-cholesterol and triglycerides, without significant changes in free fatty acids. Moreover, old rats with aCLD had higher hepatic lipid accumulation as compared to young rats analyzed by oil red-o-staining.

Interestingly, plasma levels of LPS were significantly higher in aged animals with aCLD, suggesting increased bacterial translocation in this group.

**Figure 4.**
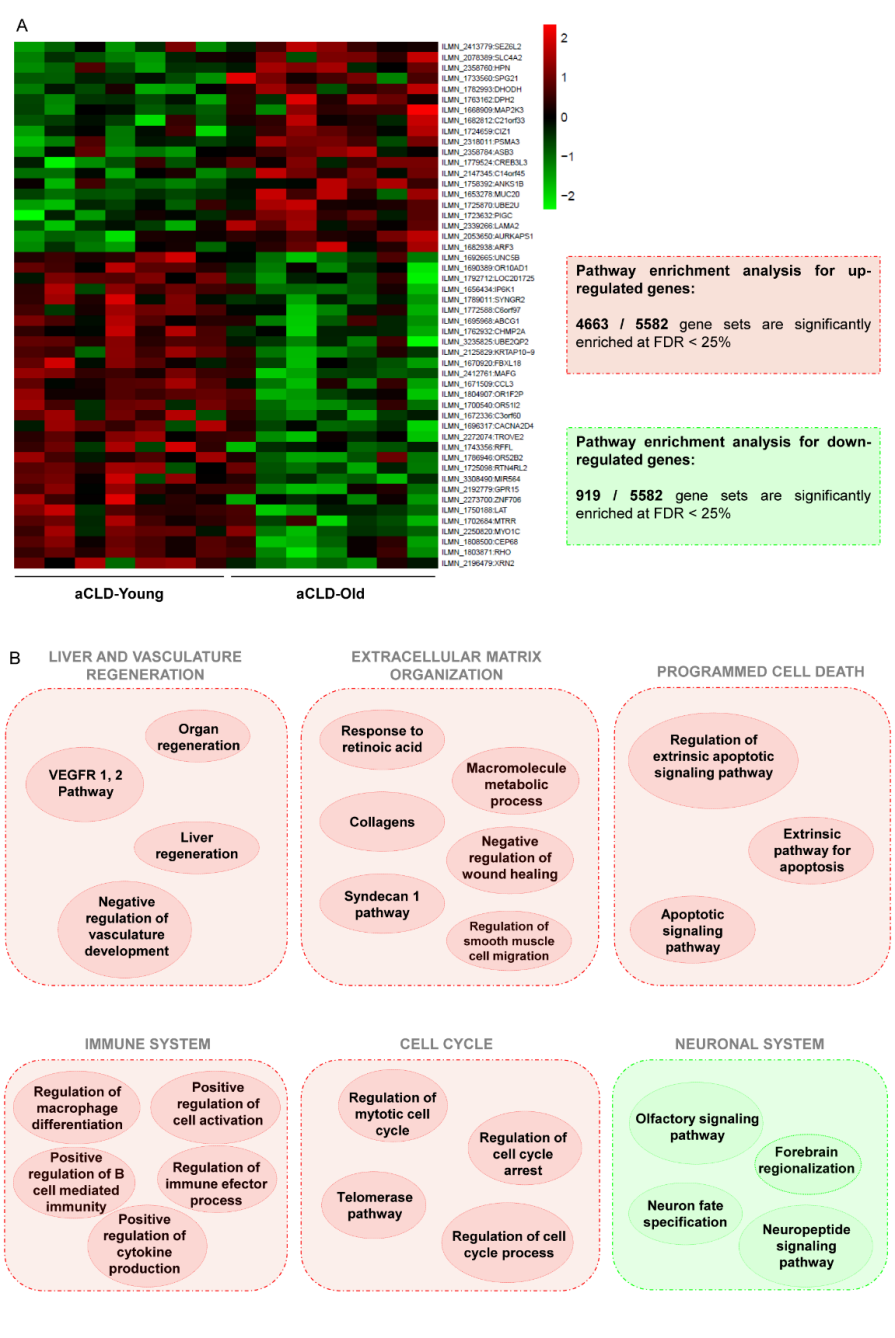
Aged-related changes in the gene signature of cirrhotic human liver Gene expression analysis in cirrhotic young and old human livers. (**A**) *Left*, fold enrichments (log_2_) are plotted in a heatmap using red colour for transcripts that are increased or using green colour for transcripts that are decreased in old cirrhotic humans. *Right*, pathway enrichment analysis results for genes upregulated (red) and downregulated (green) are summarized. (**B**) Representative gene sets upregulated (red) or downregulated (green) related to microcirculatory function in old cirrhotic humans, full description of top ten gene sets can be found in supplementary materials. FDR < 10%, n=7 per group. Clinical characteristics of donors are described in Supplementary table 1.

**Figure 5.**
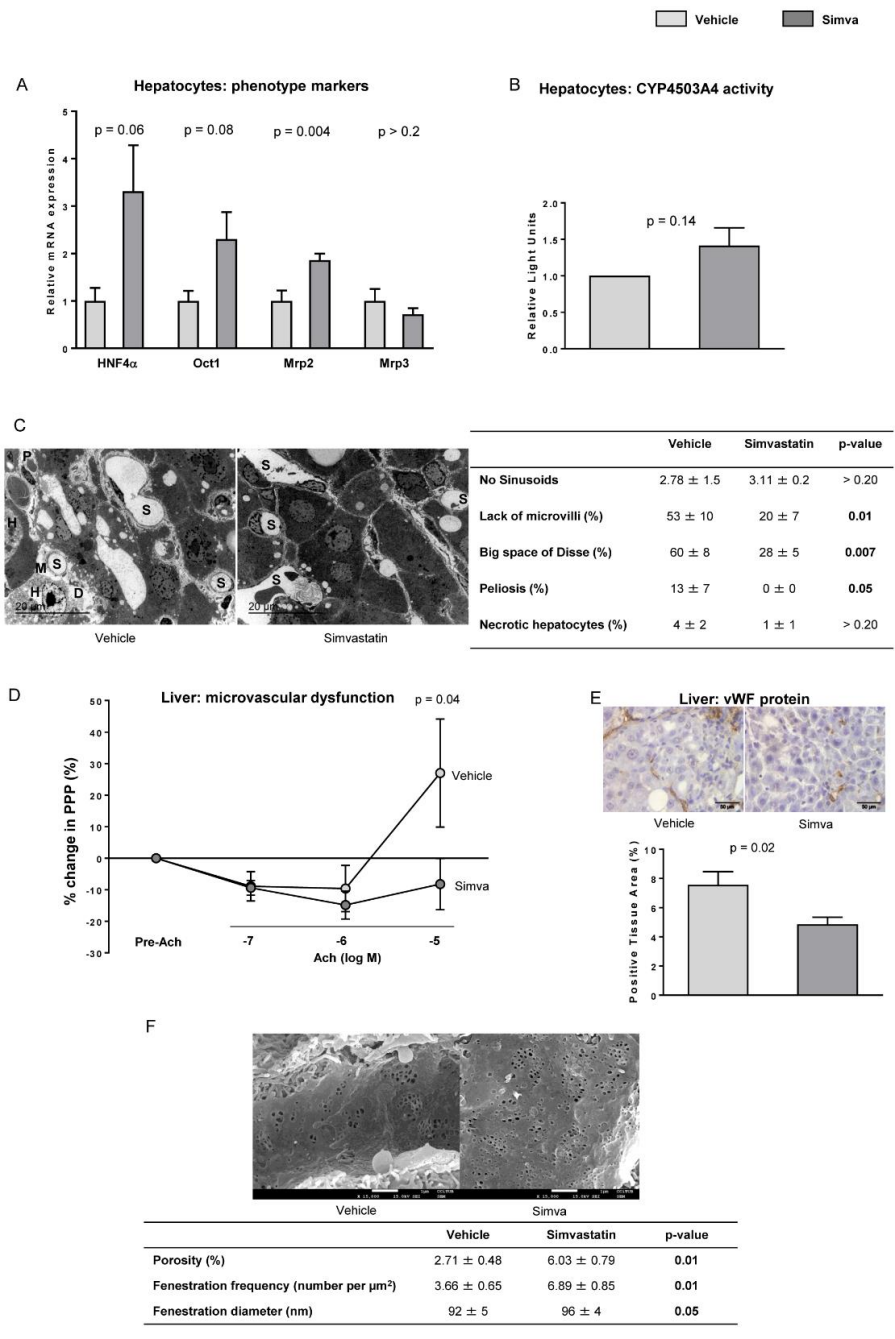
Effects of simvastatin on hepatocytes and microcirculatory function (**A**) HNF4α, Oct1, Mrp2 and Mrp3 mRNA expression in livers from aged rats with aCLD treated with simvastatin or vehicle. (**B**) Cytochrome P4503A4 activity in hepatocytes isolated from livers described in A. (**C**) Representative transmission electron microscopy images and corresponding quantification of numbers of sinusoids *(S)*, lack of microvilli *(M)*, big space of Disse *(D)*, peliosis and number of necrotic hepatocytes *(H)*. (**D**) Microvascular function evaluation in livers from aged rats with aCLD treated with simvastatin or vehicle. (**E**) Representative images of vWF immunohistochemistry and corresponding quantification from livers described in A. (**F**) Representative scanning electron microscopy images & quantification of porosity, fenestration frequency and fenestration diameter. n=10 (A-B, D-E), n=5 (B) and n=3 (C and F) per group. Results represent mean ± S.E.M. Images from C: 3000X, scale bar=20μm. Images from E: 400X, scale bar=50μm. Images from F: 15000X, scale bar=1μm.

### Portal hypertension is aggravated in aged rats with aCLD

Old animals with aCLD had significantly higher portal pressure (+18%) in comparison to young cirrhotic animals (Table 1), which was the consequence of further increases in both portal blood inflow and hepatic vascular resistance. Systemic hemodynamic parameters showed no differences between groups.

Analysis of *ex vivo* hepatic vasodilatory capacity in response to incremental concentrations of acetylcholine revealed no differences between age groups (data not shown).

### Aged rats with aCLD present deterioration in hepatocyte phenotype

Ultrastructural analysis of liver architecture using transmission electron microscopy revealed that aged cirrhotic rats exhibit greater liver injury when compared to young. Indeed, and as shown in Figure 1A, old livers with aCLD presented decreased number of sinusoids, loss of hepatocyte microvilli, greater presence of erythrocytes inside the space of Disse (peliosis), further deposition of basal lamina in the sinusoid, and a significant increase in the percentage of necrotic hepatocytes. Further analysis of hepatocyte phenotype markers in liver tissue confirmed a marked deregulation in aging (Fig 1B).

### The sinusoidal endothelium of aged rats with aCLD is further capillarized

As shown in Figure 2, the expression of a variety of vasoprotective, vasodilatory and angiocrine molecules, including the transcription factor KLF2, the LSEC-specific marker CD32b, and the anti-inflammatory and vasodilatory protein eNOS, were significantly deregulated in old cirrhotic livers in comparison to young. Additional LSEC analysis using scanning electron microscopy revealed a reduction in fenestrae porosity and frequency, altogether suggesting that aged cirrhotic rats undergo significantly greater sinusoidal capillarization.

### Aged cirrhotic animals exhibit exacerbated fibrosis and over-activation of HSC and macrophages

Analysis of extracellular matrix deposition in aged cirrhotic livers evidenced a significant increment in hepatic fibrosis, as demonstrated by increased Sirius Red staining (Fig. 3A) and collagen I protein expression (Fig. 3B). According with this result, HSC phenotype markers α-SMA and phosphorylated moesin were overexpressed in aged cirrhotic liver tissue (Fig. 3C & D), with no differences in desmin expression (data not shown), thus suggesting over-activation of this hepatic cell type. Finally, characterization of the hepatic macrophage phenotype revealed increased infiltration of CD68+ cells (Fig. 3E) with no differences in CD163+ cells (Fig. 3E), and significant differences in the mRNA expression of different cytokines including TNFα, Mrc1 and Arg1 (Fig. 3F).

**Figure 6.**
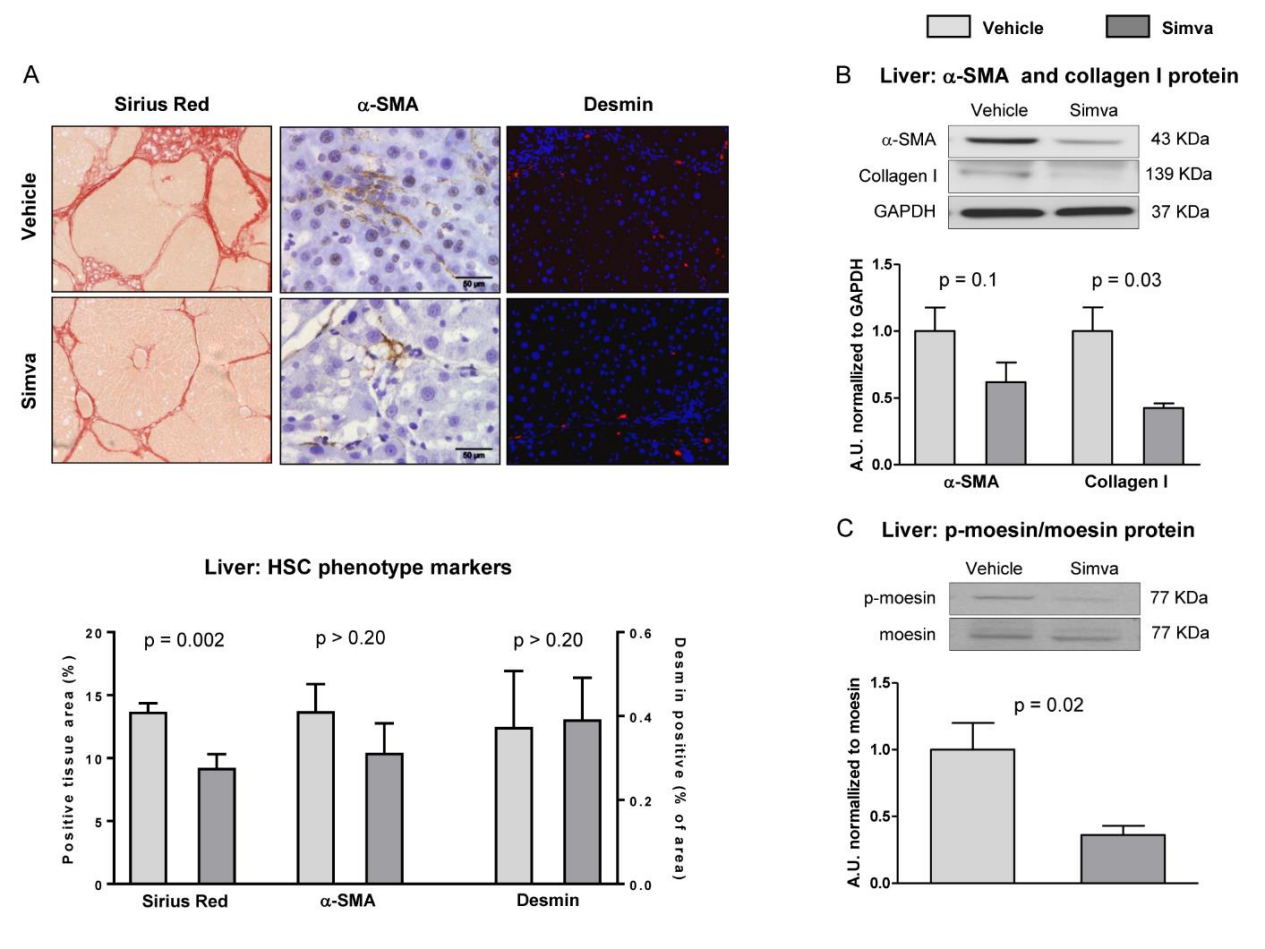
Simvastatin promotes decreased fibrosis deposition and HSC de-activation (**A**) Representative images of fibrotic content, α-SMA and desmin with their corresponding quantifications. (**B**) α-SMA and Collagen I protein expression in total liver tissue, normalized to GAPDH. (**C**) Representative western blots of moesin and p-moesin and corresponding quantification. n=10 (A-C). Results represent mean ± S.E.M. All images 400X, scale bar=50μm.

**Table 2:** Biometric, biochemical and hemodynamic characteristics in aCLD-old rats treated with simvastatin or vehicle.

	Vehicle	Simvastatin	% change	p-value
**Body weight (g)**	655 ± 31	681 ± 18	+4	> 0.20
**Liver (g)**	16.6 ± 1.0	19.1 ± 1.1	+15	0.15
**Liver-body weight ratio (%)**	2.58 ± 0.19	2.81 ± 0.15	+9	> 0.20
**AST (U/L)**	188 ± 34	155 ± 28	-18	> 0.20
**ALT (U/L)**	61 ± 5	57 ± 6	-7	> 0.20
**CKM (ng/mL)**	64 ± 13	47 ± 10	-27	> 0.20
**Bilirubin (mg/dL)**	0.23 ± 0.06	0.10 ± 0.00	-57	**0.05**
Bile production (µL/min*100g bw)	23.5 ± 7.1	42.0 ± 16.5	+45	0.17
**Albumin (mg/dL)**	22.0 ± 1.1	24.2 ± 0.8	+10	0.10
**Plasma cholesterol (mg/dL)**	86 ± 5	79 ± 10	-8	> 0.20
**Plasma LDL cholesterol (mg/dL)**	64 ± 5	55 ± 7	-14	> 0.20
**Plasma HDL cholesterol (mg/dL)**	14.0 ± 1.5	15.6 ± 2.6	+7	> 0.20
**Plasma triglycerides (mg/dL)**	38.0 ± 5.1	42.1 ± 7.1	+11	> 0.20
**Oil red O-staining (%)**	1.33 ± 0.22	1.64 ± 0.39	+23	> 0.20
**MDA (nmol/mg protein)**	2.68 ± 0.74	2.35 ± 0.30	-12	0.15
**LPS (EU/mL)**	1.49 ± 0.18	1.62 ± 0.13	+8	> 0.20

**PP (mmHg)**	15.9 ± 1.4	11.9 ± 0.8	-25	**0.02**
PBF (mL/min*g)	1.28 ± 0.17	1.13 ± 0.16	-12	>0.20
HVR (mmHg*min/mL*g)	13.9 ± 2.4	12.4 ± 1.5	-11	>0.20
*Ex vivo* HVR (mmHg*min/mL*g)	0.39 ± 0.04	0.32 ± 0.03	-18	0.20
**MAP (mmHg)**	97 ± 6	110 ± 5	+13	>0.20
**HR (bpm)**	332 ± 22	381 ± 25	+15	0.06

Data expressed as mean ± SEM (n = 10 each group). AST: aspartate transaminase; ALT: alanine transaminase; CKM: Creatinine kinase from muscle; LDL: low density lipoprotein; HDL: high density lipoprotein; MDA: malondialdehyde; LPS: lipopolysaccharide; EU: endotoxin units; PP: portal pressure; PBF: portal blood flow; HVR: hepatic vascular resistance; MAP: mean arterial pressure; HR: heart rate.

### Livers from old patients display significant variations in the gene expression signature of cirrhosis

Supplementary table 1 shows the clinical and biochemical parameters of patients with aCLD included in the microarray analysis. Interestingly, older cirrhotic patients exhibited higher hepatic venous pressure gradient (HVPG) than young, with no differences in biochemical or clinical determinations except for gender. Possible gender influence in the analysis of gene expression was prevented as described in the methods section.

Analysis of gene expression in liver tissue identified 382 genes differentially expressed comparing young and aged cirrhotic patients (Fig. 4A). 204 genes were over-expressed while 178 genes were downregulated in aging.

A more comprehensive characterization of the aged cirrhotic liver gene signature was performed using pathway enrichment analysis (PEA). PEA for upregulated genes showed 4663 gene sets significantly enriched at a false discovery rate (FDR)<25%, while 52 were significantly enriched at FDR<10%. Supplementary table 2 shows the top ten significantly upregulated pathways. Focusing on the top upregulated pathways related to vascular pathobiology, these included: negative regulation of vascular development, response to retinoic acid, collagens and regulation of macrophage differentiation (Supplementary table 3 and Fig. 4B).

PEA for downregulated pathways showed 919 gene sets significantly enriched at FDR<25%, being 4 gene sets significantly enriched at FDR<10%. Top ten significantly downregulated pathways are detailed in Supplementary table 4, and included olfactory signalling pathway and neuron fate specification, among others.

### Aged cirrhotic rats treated with simvastatin: biometric and biochemical characteristics

In comparison to vehicle, simvastatin-treated rats presented no differences in body weight, and a slight non-significant increase in liver mass (Table 2). Biochemical analysis showed improvements in bilirubin and albumin, which together with increased bile production and no modification in creatine kinase from muscle, suggested a global improvement in hepatic injury without toxic effects. No differences in plasma lipid spectrum were observed comparing both groups.

### Simvastatin improves portal hypertension in aged rats with cirrhosis

Aged rats with aCLD treated with simvastatin exhibited a significant improvement in PP (-25%), associated with slight decreases in PBF and HVR, in comparison with vehicle (Table 2). In addition, systemic hemodynamics seemed to be ameliorated.

### Simvastatin improves the hepatocyte phenotype in aged cirrhotic rats

Primary hepatocytes from old rats with aCLD treated with simvastatin showed a significant improvement in several phenotypic markers related with hepatic functionality including upregulation in HNF4α, Oct1 and Mrp2 (Fig. 5A). Additionally, the activity of cytochrome P4503A4 was slightly higher compared to hepatocytes from vehicle-treated rats (Fig. 5B). Further analysis of hepatic damage in liver tissue using transmission electron microscopy revealed a significant improvement in response to simvastatin, including maintenance of hepatocyte microvilli, a decrease in the presence of enlarged spaces of Disse, and a reduction in peliosis (Fig. 5C).

### Simvastatin improves liver endothelial dysfunction in aged rats with aCLD

Analysis of the hepatic microcirculatory function using *ex vivo* liver perfusion revealed that old cirrhotic rats treated with simvastatin exhibited a significant improvement in their endothelium-dependent vasodilatory capacity (Fig. 5D), which correlated with decreased sinusoidal expression of the capillarization marker vWF (Fig. 5E). Sinusoidal porosity as well as the number of fenestrae was significantly improved in the group of aged cirrhotic rats treated with simvastatin in comparison to those receiving vehicle (Fig. 5F). In concordance with this finding, the hepatic expression of caveolin-1, protein upregulated when fenestrae disappear [30], was significantly decreased in simvastatin-treated rats (Supplementary Fig. 1A). LSEC phenotype amelioration after simvastatin treatment was further confirmed observing up-regulation in the mRNA expression of KLF2, CD32b, Hamp and Axin2 (Supplementary Fig. 1B & C), and down-expression of CD31 (Supplementary Fig. 1D) and ICAM-1 (Supplementary Fig. 1E & F). In contrast, we did not observe differences between groups in the NO-eNOS pathway (Supplementary Fig. 2).

### Simvastatin decreases liver fibrosis and promotes deactivation of HSC

Aged rats with aCLD treated with simvastatin showed significantly lower hepatic fibrosis when compared to vehicle as demonstrated by significant reductions in Sirius Red staining and collagen I (Fig. 6A & B). Expression of the activation marker α-SMA decreased in response to statin (Fig. 6A & B), while we observed no differences in desmin marker indicating no changes in HSC abundance (Fig. 6A). HSC contractility was decreased by simvastatin, as suggested by reduced expression of p-moesin (Fig. 6C) and reduced *in vitro* contractility of primary human HSC (Supplementary Fig. 3A).

Oxidative stress, a well-known pro-fibrogenic stimulus, was attenuated both in liver tissue and primary HSC isolated from aged cirrhotic rats treated with simvastatin (Supplementary Fig. 3B - D).

### Simvastatin ameliorates the pro-inflammatory status of aged cirrhotic rat livers

As shown in Supplementary Figure 4, CD68+ macrophage infiltration was significantly reduced in simvastatin-treated rats, with no changes in CD163+ positive cells and neutrophil infiltration (data not shown). Accordingly, the mRNA expression of several cytokines was decreased in simvastatin-treated rats.

## DISCUSSION

Due to its high incidence worldwide, advanced chronic liver disease (aCLD) has become a major public health problem comparable to diabetes [4, 5]. Epidemiological data confirm that cirrhosis is more prevalent in the elderly [31], it progresses faster in this sub-group of patients [32] and importantly, that decompensation is more frequent in older patients (14%) compared to younger ones (4%) [33, 34].

In the pre-clinical scenario little is known about the impact of aging on liver diseases. Previous work suggested higher rate of fibrosis deposition in aged rats and attributed this to alterations in the immune response [9, 10]. However, this is the first study that evaluates the impact of aging on the hepatic sinusoidal milieu in a validated pre-clinical model of aCLD, focusing on the pathophysiology of the disease and its hemodynamic alterations.

The main finding of the present study is that aged animals develop a more severe form of aCLD, which can be broadly defined by poorer hepatic function and exacerbated portal hypertension, fibrosis and inflammation. Indeed, aged rats with cirrhosis exhibited serious liver damage reflected by the increase in transaminases and the reduction in serum albumin and bile production in comparison with young rats with aCLD. Interestingly, aging was also accompanied by increases in both systemic and hepatic lipid content which can be an important source of oxidative stress; a well-known detrimental factor in the pathophysiology of portal hypertension and aCLD [35]. Hepatocytes experienced profound deregulations in their phenotype and more extensive cell death, which together with increased fibrosis caused greater hepatic architecture deterioration. Indeed, increased hepatocyte necrosis could also over-stimulate HSC activation and therefore further contribute to disease exacerbation [36]. Considering the high index of cell death observed in the aged group, a caspase inhibitor may be an attractive target for the treatment of chronic liver diseases [37].

At the microcirculatory level, we describe for the first time that aged rats with aCLD show a marked aggravation in portal hypertension, which could be explained by increments in both vascular resistance and liver blood inflow. We hypothesized that the deterioration of the intrahepatic microcirculatory status is a consequence of overall deregulations in the phenotype of the main liver cells.

Although no significant differences in microvascular dysfunction were observed between young and aged cirrhotic animals, a comprehensive analysis of sinusoidal de-differentiation markers revealed alterations in LSEC phenotype including a reduction in the nitric oxide synthase pathway, depletion of angiocrine mediators and importantly, reduced porosity and frequency of fenestrae. These observations agree with previous studies reporting pseudo-capillarisation of the sinusoidal endothelium during healthy aging [3, 38], revealing that these changes are much intensified in aged livers affected by chronic injury.

In addition to LSEC, HSC play a key role modulating the hepatic vascular resistance in cirrhosis [39]. Importantly, we herein describe that old HSC undergo significant changes due to chronic liver injury, which ultimately lead to expanded deposition of extracellular matrix in comparison to young animals. Interestingly, previous pre-clinical studies suggested an increment in hepatic fibrogenesis in aging [9, 10, 40], however we describe such aggravation in a model of decompensated cirrhosis that better reflects the clinical observations [32, 41].

The role of inflammation in the progression of CLD has been widely described in the past, and recently reviewed [42], nevertheless much less is known in the context of aging [10, 40]. In our study, and in comparison, to young animals, we observe that aged cirrhotic rats exhibit further deterioration in the hepatic inflammatory phenotype as demonstrated by a significant increase in the recruitment of proinflammatory macrophages together with a decrease in the expression of pro-resolution cytokines in this cellular sub-population. The increase in the myeloid cell content in the aged cirrhotic liver may derive from the sinusoidal endothelial activation described above, and importantly, this could be another mechanism contributing to microcirculatory dysfunction and global worsening of the disease [43].

In addition and aimed at understanding the translatability of our pre-clinical discoveries, we characterized the hepatic transcriptome in two groups of cirrhotic patients of different age. Although the cohort of patients included was small, the result of these analyses confirmed the alterations observed in the aging rat model: endothelial deregulation, HSC over-activation, enhanced fibrogenesis and immune cell activation. Altogether, the pre-clinical and clinical data obtained in this study suggest that the pathophysiology of CLD is much worse, and probably involves different molecular mechanisms, when comparing aged and young individuals. Future studies, out of the scope of the present manuscript, will be required to further comprehend the impact of the unique gene expression signature of aged cirrhotic livers in the progression and regression of CLD.

Statins are HMGCoA reductase inhibitors originally designed as cholesterol-lowering drugs that over the years have been re-discovered for their pleiotropic effects on inflammation, fibrosis and endothelial function, among others [44]. Different studies have reported the benefits of statins in experimental models of young cirrhosis [12-14, 16] but none has evaluated the effect of this therapy in aged aCLD.

In this study we observed that 2-week administration of simvastatin to aged cirrhotic rats significantly improves liver microvascular dysfunction and portal hypertension, accompanied by slight ameliorations in the systemic hemodynamic and in hepatic function.

Underlying mechanisms explaining the global benefits of this vasoprotective compound revealed reduced disease severity in all major hepatic cell types. Hepatocytes exhibited an improved phenotype as suggested by ameliorations in their synthetic capacity, in blood tests and in the ultrastructural architecture. Importantly, we did not observe signs of toxicity due to the treatment, suggesting that previous evidence of simvastatin toxic effects [45] may derive from the experimental model used to induce CLD (common bile duct ligation) and/or be dose dependent. Indeed, in our study we selected a dose of 5 mg/day/kg in rats, which is equivalent to a dose of 80 mg/day in adult humans following Reagan-Shaw et al. dose conversion [46], and preferred to generate CLD by chronic CCl_4_ with no obstruction of bile secretion, thus preventing drug accumulation.

As expected, simvastatin improved the phenotype of sinusoidal cells. The hepatic endothelium increased the expression of functional and angiocrine markers, while decreased the expression of capillarisation and inflammatory markers. Remarkably, aged cirrhotic animals treated with simvastatin for 15 days exhibited double number and frequency of fenestrae, which in fact may contribute to improve parenchymal function through a better diffusion of oxygen, nutrients, and waste products. It is important to denote that no previous study has demonstrated an improvement in sinusoidal porosity in response to statin administration *in vivo*, just one study showed amelioration in the fenestrae of the mesenteric endothelium in a rat model of arterial hypertension [47]. Although our results support that the hepatic endothelium was ameliorated due to the treatment, we could not detect differences in the nitric oxide pathway when comparing both groups of animals. Considering that we tested the effects of 15-day simvastatin, treatment period longer than in any previously published study, we cannot disregard the possibility that a partial spontaneous recovery in the vehicle-treated group may have occurred and consequently no differences in certain markers could be detected. Likewise, we cannot discard that aged rats with cirrhosis may exhibit a poor NO-signaling response to statins.

Importantly, simvastatin treatment promoted a marked regression in liver fibrosis, which may be due to deactivation of HSC, together with a reduction in oxidative stress and inflammation. Additionally, HSC phenotype improvement could also contribute to the amelioration in hepatic microvascular dysfunction. Our observations are in agreement with previous clinical observations in patients with liver disease [44].

In conclusion, this study provides evidence that aCLD has a much-aggravated pathophysiology in aged individuals, and even more importantly, that aging may activate different or additional molecular mechanisms from those observed in young. Considering the increasing age of many patients with aCLD, we propose that using closer to real-life models to investigate the pathophysiology of aCLD may allow the development of more reliable therapeutic strategies. In fact, the characterization of simvastatin in this model further recommends its applicability at the bedside.

## Supplemetary Materials

The Supplemenantry data can be found online at: www.aginganddisease.org/EN/10.14336/AD.2019.0127.


