# Supplementary material for: Aging Influences Hepatic Microvascular Biology and Liver Fibrosis in Advanced Chronic Liver Disease
Source: Aging Dis. 2019 Aug 1;10(4):684–98. doi: 10.14336/AD.2019.0127 (PMC6675529; doi:10.14336/AD.2019.0127)
Supplement: Supplementary file 1 [file AD-10-4-684-s.pdf]

SUPPLEMENTARY DATA

Aging Influences Hepatic Microvascular Biology and Liver Fibrosis in Advanced Chronic Liver Disease

Raquel Maeso-Díaz[1], Martí Ortega-Ribera[1], Erica Lafoz[1], Juan José Lozano[2], Anna Baiges[1,2], Rubén Francés[2,3], Agustín Albillos[2,4], Carmen Peralta[2,5], Juan Carlos García-Pagán[1,2], Jaime Bosch[1,2,6], Victoria C Cogger[7], Jordi Gracia-Sancho[1,2,6,*]

[1]Liver Vascular Biology Research Group, Barcelona Hepatic Hemodynamic Laboratory, IDIBAPS Biomedical Research Institute, University of Barcelona Medical School, Barcelona, Spain
[2]Biomedical Research Network Center in Hepatic and Digestive Diseases (CIBEREHD), Madrid, Spain
[3]Instituto de Investigación Sanitaria y Biomédica de Alicante (ISABIAL -Fundación FISABIO), Alicante, Spain
[4] Department of Gastroenterology and Hepatology, Hospital Universitario Ramón y Cajal, IRYCIS, Universidad de Alcalá, Madrid, Spain
[5]Protective Strategies Against Hepatic Ischemia-Reperfusion Group, IDIBAPS, Barcelona, Spain
[6]Hepatology, Department of Biomedical Research, Inselspital, Bern University, Switzerland
[7]Centre for Education and Research on Ageing & ANZAC Research Institute, University of Sydney and Concord Hospital, Sydney, Australia

© 2019. Maeso-Díaz R et al. Published online at http://www.aginganddisease.org/EN/10.14336/AD.2019.0127

SUPPLEMENTARY DATA

Supplementary materials and methods

In vivo hemodynamic

Rats were anesthetized with ketamine (100 mg/kg body weight, Imalgene 1000; Merial) plus midazolam (5 mg/kg body weight; Laboratorio Reig Jofre, S.A., Spain) intraperitoneally, fastened to a surgical board, and maintained at a constant temperature of 37 ± 0.5ºC.

A tracheotomy and cannulation with a PE-240 catheter (Portex) was performed in order to maintain adequate respiration during anesthesia. Indwelling catheters made of polyethylene tubing (PE-50; Portex, UK) were placed into the femoral artery to measure mean arterial pressure (MAP; mm Hg) and heart rate (HR; beats per minute), and to the ileocolic vein to measure PP (mmHg). PBF (mL/min) was measured with a nonconstrictive perivascular ultrasonic transit-time flow probe (2PR, 2-mm diameter; Transonic Systems Inc., USA) placed around the portal vein just before its entrance in the liver, avoiding the measurement of most portal-collateral blood flow. The flow probe and pressure transducers were connected to a Powerlab (4SP) linked to a computer using Chart v5.5.6 for Windows software (AD Instruments, Australia). Hepatic vascular resistance (HVR) was calculated as PP/PBF. Hemodynamic data were collected after a 20-minute stabilization period [1]. At the end of the *in vivo* hemodynamic study, plasma samples from young and aged-rats were collected to subsequently evaluate alanine aminotransferase (ALT), aspartate aminotransferase (AST), bilirubin, albumin, cholesterol, low density lipoprotein cholesterol (LDL), high density lipoprotein cholesterol (HDL), triglycerides and free fatty acids (FFA), all by standard protocols. Experiments and data collection were performed blindly.

Liver endothelial function

Rat livers were isolated and perfused at 35 mL/min with Krebs' buffer. The perfused rat liver preparation was allowed to stabilize for 20 min before vasoactive substances were added. The intrahepatic microcirculation was pre-constricted by adding the α1-adrenergic agonist methoxamine (Mtx; 10^{-4}M; Sigma) to the reservoir. After 5 min, concentration-response curves to cumulative doses of acetylcholine (Ach; 10^{-7}-10^{-5}M; Sigma) were evaluated. The concentration of Ach was increased by 1log unit every 1.5 min interval. Responses to Ach were calculated as the percentage change in portal perfusion pressure [2]. The gross appearance of the liver, stable perfusion pressure, bile production over 0.4µl/min/g of liver and stable buffer pH (7.4 ± 0.5) were monitored during this period. If any viability or stability criteria were not satisfied, the experiment was discarded.

Hepatic cells isolation

Hepatocytes, Kupffer cells (KC) and Liver Sinusoidal Endothelial Cells (LSEC) were isolated using a well-established protocol [1]. Rat livers were perfused through the portal vein with Hanks without Ca^{+2} and Mg^{+2} containing 12 mM hepes (H3375, Sigma) pH 7.4, 0.6 mM ethylene glycol-bis (2-aminoethylether)-N,N,N′,N′-tetra acetic acid (E4378, Sigma) and 0.23 mM bovine serum albumin (BSA; A1391,0100, Applichem). Then, perfused for 30 min with 0.015% collagenase A (103586, Roche) Hanks containing 12 mM hepes (pH 7.4) and 4 mM $CaCl_2$. The resultant digested liver was excised, and *in vitro* digestion was performed at 37°C with 0.01% collagenase A, Hank's containing 12 mM hepes (pH 7.4) and 4 mM $CaCl_2$ for 10 min. Disaggregated tissue was filtered using 100 µm nylon strainer, collected in cold Krebs' buffer and centrifuged at 50 g for 5 min. The pellet was washed three times for hepatocytes enrichment. Hepatocytes were seeded in collagen-coated wells and cultured in Dulbecco's Modified Eagle's Medium (DMEMF12; 11320074, Gibco) supplemented with 2% fetal bovine serum (04-001-1A, Reactiva), 1% L-glutamine (25030-024, Gibco), 1% penicillin plus 1% streptomycin (03-331-1C, Reactiva), 1 nM dexamethasone (D4902, Sigma), 1 µM insulin (103755, HCB) and 1% amphotericin B (03-029-1C, Reactiva). The supernatant was centrifuged at 800g for 10 min and the obtained pellet was resuspended in Dulbecco's PBS (DPBS) and centrifuged through a two-step Percoll gradient (25-50%). The interface of the gradient was enriched in KC and LSEC. This cell fraction was resuspended in RPMI medium, seeded in plastic dishes and incubated for 30 min at 37ºC in humid atmosphere with 5% CO_2 in order to enhance KC purity. Non-adherent cells were seeded in collagen-coated wells and incubated for 1h (37ºC, 5% CO_2). After this time the medium was discarded and LSEC adhered cells were washed twice with DPBS and cultured in RPMI-1640 (01-100-1A, Reactiva) supplemented with 10% fetal bovine serum (04-001-1A, Reactiva), 1% L-glutamine (25030-024, Gibco), 1% penicillin plus 1% streptomycin (03-331-1C, Reactiva), 0.1 mg/mL heparin (H3393, Sigma),

© 2019. Maeso-Díaz R et al. Published online at http://www.aginganddisease.org/EN/10.14336/AD.2019.0127

0.05 mg/mL endothelial cell growth supplement (BT-203, BT) and 1% amphotericin B (03-029-1C, Reactiva). HSC were isolated through a sequential in situ perfusion of the liver with 0.195 mg/mL collagenase A (Roche), 1.5 mg/mL pronase (Roche) and 0.05 mg/ml Dnase (Roche) in Gey's Balanced Salt Solution (GBSS; Sigma), and dispersed cells were fractionated by density gradient centrifugation using 11.5% Optiprep (Sigma).[2] HSC were cultured in Iscove's Modified Dulbecco's Media (IMDM, Invitrogen, Gibco) supplemented with 10% fetal bovine serum (04-001-1A, Reactiva), 1% L-glutamine (25030-024, Gibco), 1% penicillin plus 1% streptomycin (03-331-1C, Reactiva) and 1% amphotericin B (03-029-1C, Reactiva). Viability and purity were systematically over 95%.

Electron microscopy

Liver sinusoidal ultrastructure was characterized using electron microscopy as previously described [3]. Briefly, livers were perfused through the portal vein with a fixation solution containing 2.5% glutaraldehyde and 2% paraformaldehyde in 0.1M cacodylate buffer 0.1% sucrose and fixed overnight at 4ºC. Samples were washed 3 times with 0.1M sodium cacodylate buffer. Liver sections were post-fixed with 1% osmium in cacodylate buffer and dehydrated in an ethanol gradient to 100%.

For scanning electron microscopy 6 to 8 liver blocks per sample were mounted on stubs, sputter coated with gold and examined using a Jeol 6380 scanning electron microscope. Measurements of fenestrae size, number and density were carried out. Fenestrations were defined as open pores with diameters <300nm. Diameter was defined as the major length of each fenestration or gap. Porosity was defined as the sum area of fenestrations/total quantified area. Frequency was defined as number of fenestrae per μm^2. At least 10 images per animal were taken.

For transmission electron microscopy, fixed liver tissue was embedded in Spurr resin, cut in 50nm ultrathin sections, counterstained with uranyl acetate and lead citrate and examined using microscope. 10 micrographs per sample were taken to estimate % of necrotic hepatocytes and % of sinusoids presenting each of the following parameters: lack of microvilli, big space of Disse, peliosis and basal lamina deposition.

RNA isolation and quantitative PCR

RNA from cells and tissue were extracted using RNeasy mini kit (Qiagen) and Trizol (Life Technologies), respectively. RNA quantification was performed using a NanoDrop spectrophotometer. cDNA was obtained using QuantiTect reverse transcription kit (Qiagen). Real-Time PCR was performed in an ABI PRISM 7900HT Fast Real-Time PCR System, using TaqMan predesigned probes for HNF4α (Rn04339144_m1), Oct1 (Rn00562250_m1), Mrp2 (Rn00563231_m1), Mrp3 (Rn01452854_m1), eNOS (Rn02132634_s1), CD31 (Rn01467262_m1), KLF2 (Rn01420495_g1), CD32b (Rn00598391_m1), α-SMA (Rn01759928_g1), PDGFRβ (Rn01491838_m1), TNF-α (Rn01525859_g1), iNOS (Rn00561646_m1), IL-6 (Rn01410330_m1), Mrc1 (Rn01487342_m1), Arg1 (Rn00691090_m1), IL-10 (Rn00563409_m1), HGF (Rn00566673_m1), Wnt2 (Rn01500736_m1), Hamp (Rn00584987_m1), Axin2 (Rn00577441_m1)and GAPDH (Rn01775763_g1) as endogenous controls. Results, expressed as $2^{-\Delta\Delta Ct}$, represent the x-fold increase of gene expression compared with the young group.

Western Blotting

Liver samples were processed and western blot performed as described [4]. Used primary antibodies: phosphorylated eNOS at Ser1177 (9571, Cell Signaling), total eNOS (610297, BD Transduction Laboratories), phosphorylated moesin at Thr558 (sc-12895, Santa Cruz), total moesin (sc-13122, Santa Cruz), caveolin-1 (610059, BD Biosciences), α-SMA (A2547, Sigma Aldrich), CD31 (555027, BD Biosciences), collagen I (84336, Cell Signalling) and ICAM-1 (AF583, R&D Systems) all 1:500. Blots were revealed by chemiluminescence and protein expression was determined by densitometric analysis using the Science Lab 2001 Image Gauge (Fuji Photo Film, Düsseldorf, Germany). Blots were also assayed for GAPDH (1:1000, Sigma-Aldrich) content as standardization of sample loading.

Histological Analysis

Liver samples were fixed in 10% formalin, embedded in paraffin, sectioned, and slides were stained with Sirius Red for liver fibrosis evaluation [5].
Frozen sections were cut to 10 µm and stained with Oil Red O (Sigma Aldrich) for lipid analysis. Lipid droplets were evaluated as the red-stained area per total area using ImageJ software.

© 2019. Maeso-Díaz R et al. Published online at http://www.aginganddisease.org/EN/10.14336/AD.2019.0127

SUPPLEMENTARY DATA

Immunohistochemistry

Liver samples were fixed in 10% formalin, embedded in paraffin, sectioned and processed for immunohistochemistry (IHC) or immunofluorescence (IF) as previously described [6].

For IHC liver sections were incubated with antibodies against CD32b (sc-13271, Santa Cruz), eNOS (sc-654, Santa Cruz), vWF (A0082, Dako), α-SMA (M0851, Dako), or CD163 (MCA342R, Biorad). After incubation with corresponding secondary antibodies, color development was induced by incubation with a DAB kit (Dako) and counterstained with hematoxylin. Sections were dehydrated and mounted. The specific staining was visualized and fifteen images per liver were acquired using a microscope equipped with a digital camera and the assistance of Axiovision software. The relative volume was calculated by dividing the number of points positive in sinusoidal areas by the total number of points over liver tissue [7].

For IF, liver sections were incubated with antibodies against desmin (M0760, Dako) and CD68 (MCA341R, Biorad), incubated with secondary antibodies Alexa Fluor 488 or 555 (1:400, Life technologies) and 4′,6-diamino-2-fenilindol (1:3000; DAPI, Sigma-Aldrich) and mounted in Fluoromount G medium. Ten images per sample were obtained with a fluorescence microscope and percentage of positive area (desmin) or positive cells per field (CD68) were quantified.

Cytochrome 4503A4 activity

Phase I detoxification capacity of hepatocytes was analyzed using P450-Glo™ CYP4503A4 Assay following manufacturer's instructions (V8901, Promega) [8]. Briefly, hepatocytes cultured for 24 hours were rinsed twice with DPBS and incubated with culture media containing 50 µM Luciferin-PFBE at 37°C for 4h. Then supernatant was collected and neutralized with Luciferin Detection Reagent. After incubation for 30 min at RT, plate luminescence was read in a luminometer (Orion II Microplate Luminometer, Germany). Samples luminescence was corrected subtracting background luminescence.

Human HSC isolation and contraction assay

Human hepatic stellate cells were isolated from remnant liver tissue approximately weighting 20 g obtained from discarded tissue after liver transplantation (chronic ethanol etiology). Ethics Committee of the Hospital Clínic de Barcelona approved the experimental protocol (HCB/2015/0624) and in all cases patients received and agreed informed consent. Median age of liver donors for cell isolation was 70 ± 3 years.

Briefly, livers were perfused through major vessels for 10 min with Hanks without Ca^{+2} and Mg^{+2} containing 12 mM hepes (H3375, Sigma) pH 7.4, 0.6 mM ethylene glycol-bis (2-aminoethylether)-N,N,N′,N′-tetra acetic acid (E4378, Sigma) and 0.23 mM bovine serum albumin (BSA; A1391,0100, Applichem). Then, perfused for 30 min with 0.015% collagenase A (103586, Roche) Hanks containing 12 mM hepes (pH 7.4) and 4 mM $CaCl_2$. The resultant digested liver was excised and *in vitro* digestion was performed at 37°C with 0.01% collagenase A, Hank's containing 12 mM hepes (pH 7.4) and 4 mM $CaCl_2$ for 10 min. Disaggregated tissue was filtered using 100 µm nylon strainer, collected in cold Krebs buffer and centrifuged at 50 g for 5 min. Hepatocytes were contained in the pellet while non-parenchymal cells were found in the supernatant. HSC were fractionated by density gradient centrifugation using 11.5% Optiprep (Sigma) as previously described [8]. HSC were cultured in Iscove's Modified Dulbecco's Media (IMDM, Invitrogen, Gibco) supplemented with 10% fetal bovine serum (04-001-1A, Reactiva), 1% L-glutamine (25030-024, Gibco), 1% penicillin plus 1% streptomycin (03-331-1C, Reactiva) and 1% amphotericin B (03-029-1C, Reactiva). Viability and purity were systematically over 95%.

Contraction of aged human HSC was performed as previously described [2]. Culture plates were incubated with 1% BSA-PBS and afterwards filled with a mix of collagen (2 mg/mL) and human HSC ($1–2 \times 10^5$ cells/mL). Once the gels were solidified, serum free IMDM with 10 µM simvastatin or vehicle was added. After 24 h, contraction was induced by adding 10% FBS for 24 h. Finally, the contraction area was digitalized and measured with ImageJ software. The results are expressed as % of contraction relative to the initial area of the gel.

Endotoxemia quantification

Endotoxemia was quantified using a quantitative chromogenic limulus amoebocyte lysate (LAL) test (BioWhittaker, Nottingham, UK), following manufacturer's instructions. Due to LPS ubiquity, samples and reagents were handled in

an airflow chamber and processed with pyrogen-free material tested by manufacturers. E. coli lyophilized endotoxin (22 UE/mL) provided by the kit was used to set standard endotoxin concentrations ranging from 5.0 UE/mL to 0.1 UE/mL. To verify the lack of product inhibition by plasma protein, a dilution/heating inactivation protocol was followed prior to endotoxin measurement. A pooled E. coli endotoxin spike solution (0.4 UE/mL) was prepared with serum samples. Dilutions ranging from 1/2 to 1/20 were performed over spiked and unspiked serum samples. All test samples were then incubated at 60°C during 30 min [9]. The LAL test was performed after this period. The non-inhibitory dilution was established when the difference between spiked and unspiked endotoxin values was equal to the known concentration of the spike ± 25%, as detailed by the manufacturer. Final sample dilutions used were 1/10 (spike recovery after correction of dilution: 0.34 UE/mL). All samples were tested in triplicate and read at 405 nm in a Tecan Sunrise microplate reader (Männedorf, Switzerland).

Supplementary references

[1] Gracia-Sancho J, Laviña B, Rodríguez-Vilarrupla A, Brandes RP, Fernández M, Bosch J, et al. (2007). Evidence Against a Role for NADPH Oxidase Modulating Hepatic Vascular Tone in Cirrhosis. Gastroenterology, 133: 959–66.

[2] De Mesquita FC, Guixé-Muntet S, Fernández-Iglesias A, Maeso-Diaz R, Vila S, Hide D, et al. (2017). Liraglutide improves liver microvascular dysfunction in cirrhosis: Evidence from translational studies. Sci Rep, 7: 1–10.

[3] Le Couteur DG, Cogger VC, Markus AMA, Harvey PJ, Yin ZL, Ansselin AD, et al. (2001). Pseudocapillarization and associated energy limitation in the aged rat liver. Hepatology, 33: 537–43.

[4] Guixe-Muntet S, de Mesquita FC, Vila S, Hernandez-Gea V, Peralta C, Garcia-Pagan JC, et al. (2016). Cross-talk between autophagy and KLF2 determines endothelial cell phenotype and microvascular function in acute liver injury. J Hepatol, 66: 86-94.

[5] Gracia-Sancho J, Russo L, García-Calderó H, García-Pagán JC, García-Cardeña G, Bosch J (2011). Endothelial expression of transcription factor Kruppel-like factor 2 and its vasoprotective target genes in the normal and cirrhotic rat liver. Gut, 60: 517–24.

[6] Marrone G, Maeso-Díaz R, García-Cardena G, Abraldes JG, García-Pagán JC, Bosch J, et al. (2015). KLF2 exerts antifibrotic and vasoprotective effects in cirrhotic rat livers: Behind the molecular mechanisms of statins. Gut, 64: 1434–43.

[7] Vilaseca M, García-Calderó H, Lafoz E, Ruart M, López-Sanjurjo CI, Murphy MP, et al. (2017). Mitochondria-targeted antioxidant mitoquinone deactivates human and rat hepatic stellate cells and reduces portal hypertension in cirrhotic rats. Liver Int, 37: 1002–12.

[8] Ortega-Ribera M, Fernández-Iglesias A, Illa X, Moya A, Molina V, Maeso-Díaz R, et al. (2018). Resemblance of the human liver sinusoid in a fluidic device with biomedical and pharmaceutical applications. Biotechnol Bioeng, 115: 2585-2594.

[9] Roth RI, Levin FC, Levin J (1990). Optimization of detection of bacterial endotoxin in plasma with the Limulus test. J Lab Clin Med, 116: 153-61.

© 2019. Maeso-Díaz R et al. Published online at http://www.aginganddisease.org/EN/10.14336/AD.2019.0127

SUPPLEMENTARY DATA

Supplementary table 1. Clinical characteristics of patients with liver cirrhosis included in the microarray analysis.

	**Young patients (age < 50 years)**	**Old patients (age > 50 years)**	**p-value**
Age (years)	42 ± 5.5	62 ± 4.1	**0.00**
Male	6 (86%)	1 (14%)	**0.01**
Cirrhosis etiology naive HCV	5 (71%)	5 (71%)	
treated HCV	2 (29%)	0	> 0.2
HCV + OH	0	1 (14%)	
cryptogenic	0	1 (14%)	
Child Pugh class A/B/C	7/0/0	4/3/0	0.19
MELD	8.5 ± 2.3	10.0 ± 3.8	> 0.2
Ascites	0	3 (42%)	0.19
Hepatic encephalopathy	0	1 (14%)	> 0.2
Gastroesophageal varices	3 (42%)	5 (71%)	> 0.2
Variceal bleeding	0	2 (29%)	> 0.2
HVPG (mmHg)	11.3 ± 4.4	16.3 ± 3.9	**0.03**
Bilirubin (mg/dL)	1.1 ± 0.7	2.1 ± 2.2	> 0.2
AST (U/L)	110.5 ± 64.0	127.8 ± 114.8	> 0.2
ALT (U/L)	116.0 ± 70.0	80.8 ± 65.3	> 0.2
Albumin (mg/dL)	42.3 ± 3.0	37.5 ± 9.1	0.13
Prothrombin time (%)	77.5 ± 6.4	81.3 ± 13.2	> 0.2

Data expressed as mean ± SD (n=7 each group).

© 2019. Maeso-Díaz R et al. Published online at http://www.aginganddisease.org/EN/10.14336/AD.2019.0127

SUPPLEMENTARY DATA

Supplementary table 2. Pathway enrichment analysis for genes up-regulated in aged cirrhotic patients.

Pathway	No. Genes up-regulated from total gene set	Genes	Database
Positive regulation of viral process	44/82	VPS4B, HPN, GTF2F1, LGALS1, ADARB1, SMARCA4, CDK9, CD28, TRIM11, SMARCB1, …	GO
Positive regulation of multiorganism process	68/139	VPS4B, HPN, GTF2F1, LY86, PLAU, LGALS1, ADARB1, SMARCA4, CDK9, ACVR1B, …	GO
VEGFR1,2 Pathway	36/63	MAP2K3, PTPN6, ARF1, NOS3, MAPK14, CDC42, PRKAB1, MAPK3, GRB10, AKAP1, …	PID
Endomembrane system organization	154/403	TULP1, RAB8A, ARL1, CLASP1, VPS4B, PVRL2, RPR, ZNF385A, VPS33B, RAB34, …	GO
Extrinsic apoptotic signalling pathway via death domain receptors	17/34	DAB2IP, NGF, MOAP1, SORT1, FASLG, CRADD, DDX47, TNFRSF10B, TNFRSF1A, CASP8, …	GO
Alpha beta pathway	9/25	MAP2K3, HCK, MAPK14, CDC42, PAK1, LCK, FYN, MAP2K4, RAC1, SRC	PID
Mitotic prometaphase	41/72	CLASP1, CENPH, NUP85, CCDC99, XPO1, CDC20, NUP133, NUP37, RCC2, CKAP5, …	Reactome
CXCR4 Pathway	43/93	VPS4B, HCK, PTPN6, GNAI3, INPP5D, ITGA2, CDC42, CD247, RGS1, PAK1, …	PID
Regulation of viral release from host cell	15/27	VPS4B, TRIM26, PML, TRIM11, VPS4A, CHMP4B, TRIM21, VAPA, CAV2, TSG101, …	GO
Negative regulation of extrinsic apoptotic signaling pathway via death domain RECE	16/30	NOS3, ICAM1, GPX1, SERPINE1, FASLG, RFFL, TNFRSF10B, TNFSF10, CASP8, FGG, …	GO

PID: pathway interaction database; GO: gene ontology.
Data expressed at FDR < 10% (n=7 each group).

© 2019. Maeso-Díaz R et al. Published online at http://www.aginganddisease.org/EN/10.14336/AD.2019.0127

SUPPLEMENTARY DATA

Supplementary table 3. Pathway enrichment analysis for genes up-regulated in aged cirrhotic patients. Top ten gene sets related to vascular biology.

Pathway	No. Genes up-regulated from total gene set	Genes	Database
VEGFR1,2 Pathway	36/63	MAP2K3, PTPN6, ARF1, NOS3, MAPK14, CDC42, PRKAB1, MAPK3, GRB10, AKAP1, ...	PID
Negative regulation of vasculature development	37/69	DAB2IP, THBS2, CXCL10, SPARC, DCN, PDE3B, PTGER4, SEMA4A, SERPINE1, XDH, ...	GO
Response to retinoic acid	37/95	TNC SP100 RET RNF2 CLK2 SOX9 CD38 FZD4 WNT2 ADNP2, …	GO
Collagens	21/37	COL7A1 COL6A2 COL16A1 COL1A2 COL5A1 COL1A1 COL8A2 COL5A2 COL11A1 COL9A2, …	Matrisome
Regulation of wound healing	59/114	CLASP1 PPAP2B VPS33B TSPAN8 ENPP4 PLAU NOS3 STX2 APCS HBEGF, …	GO
Regulation of smooth muscle cell migration	27/48	PLAU ITGA2 RPS6KB1 PTGER4 TACR1 SERPINE1 TPM1 P2RY6 F3 DOCK7, …	GO
Platelet derived growth factor receptor signaling pathway	21/31	BCR PLEKHA1 RAPGEF1 JAK2 PDGFRA GAB1 PIK3C2A IQGAP1 PTEN PDGFRB, …	GO
Positive regulation of the transforming growth factor beta production	6/14	CX3CL1 LUM LGALS9 CD46 THBS1 CD34	GO
Regulation of macrophage differentiation	12/19	CALCA C1QC PRKCA CASP8 CSF1 TRIB1 FADD RB1 ID2 INHA, …	GO
Positive regulation of cytokine production involved in immune response	11/29	FCER1G TRIM6 SEMA7A F2RL1 TNFSF4 B2M RSAD2 MALT1 CD74 CD36, …	GO

Data expressed at FDR < 10% (n=7 each group)
PID: pathway interaction database; GO: gene ontology.

© 2019. Maeso-Díaz R et al. Published online at http://www.aginganddisease.org/EN/10.14336/AD.2019.0127

SUPPLEMENTARY DATA

Supplementary table 4. Pathway enrichment analysis for genes down-regulated in aged cirrhotic patients.

Pathway	No. Genes down-regulated from total gene set	Genes	Database
Olfactory signaling pathway	128/282	OR9Q2 OR10AD1 OR10A3 OR51I2 OR10G9 OR4C3 OR4A5 OR5I1 OR52L1 OR5D14, …	Reactome
Olfactory transduction	158/341	PRKX OR52B2 OR9Q2 OR10AD1 OR10A3 OR51I2 OR10G9 OR4C3 OR6C74 OR4A5, …	KEGG
Sensory perception of chemical stimulus	164/405	OR52B2 OR9Q2 OR10AD1 OR10A3 OR51I2 OR5H15 SCNN1D TAS2R46 OR10G9 OR4C3, …	GO
Forebrain regionalization	15/21	SIX3 GSX2 PAX6 EOMES WNT1 EMX1 WNT7B FGF8 BMP2 BMP4, …	GO
Ventral spinal cord inter neuron differentiation	8/15	DMRT3 NKX6-1 NKX2-2 PAX6 NKX6-2 GLI2 DLL4 FOXN4	GO
Reactome FGFR1 ligand binding and activation	9/13	FGF10 FGF3 FGF9 FGF1 FGF8 FGF4 FGF17 FGF6 FGF22	Reactome
Neuron fate specification	9/24	OLIG3 DMRT3 NKX6-1 ISL1 GSX2 NKX2-2 PAX6 NKX6-2 GLI2	GO
Neuropeptide signaling pathway	35/79	SCG5 GPR139 NPW CARTPT NMUR1 NTSR2 MC2R NPVF GALR1 PPY, …	GO
Gamma Aminobutyric acid signaling pathway	10/17	PLCL1 GABRR2 GABRG3 CACNA1A GABRA5 GABRA2 GABRR1 PLCL2 ATF4 GABBR2	GO
Negative regulation of megakaryocyte differentiation	8/14	HIST2H4B HIST1H4C HIST1H4A HIST1H4B HIST1H4E HIST1H4F CIB1 HIST2H4A	GO

KEGG: Kyoto encyclopedia of genes and genomes; GO: gene ontology.
Data expressed at FDR <25% (n=7 each group).

SUPPLEMENTARY DATA

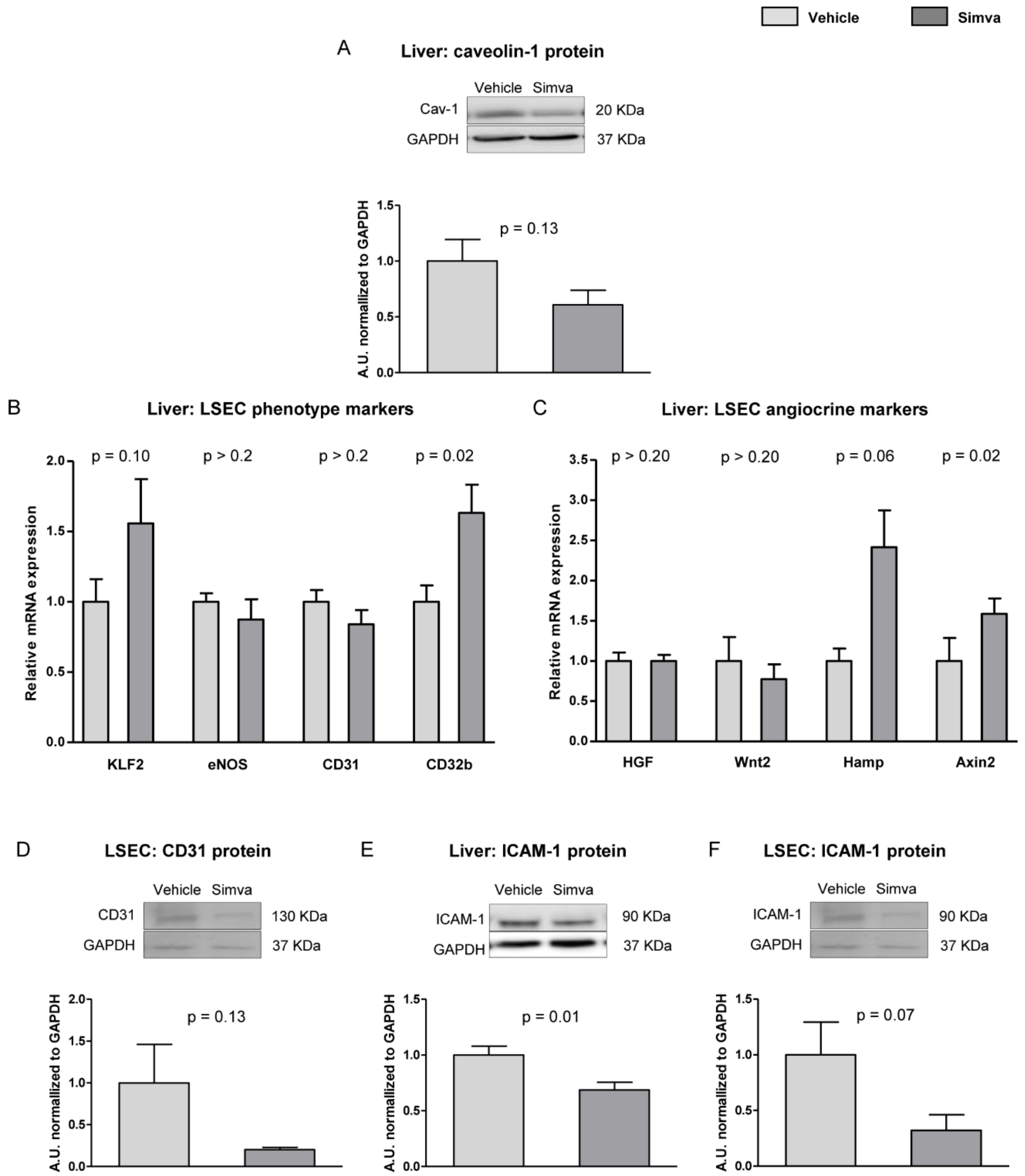

Supplementary Figure 1. Simvastatin ameliorates LSEC phenotype and microcirculatory dysfunction. (A) Caveolin-1 protein expression in total liver tissue, normalized to GAPDH. **(B)** mRNA expression of KLF2, eNOS, CD31 and CD32b. **(C)** mRNA expression of HGF, Wnt2, Hamp and Axin2. **(D)** CD31 protein expression in freshly isolated LSEC, normalized to GAPDH. **(E)** ICAM-1 protein expression in total liver tissue, normalized to GAPDH. **(F)** ICAM-1 protein expression in LSEC, normalized to GAPDH. n=10 (A-C, E) and n=5 (D, F) per group. Results represent mean ± S.E.M.

© 2019. Maeso-Díaz R et al. Published online at http://www.aginganddisease.org/EN/10.14336/AD.2019.0127

SUPPLEMENTARY DATA

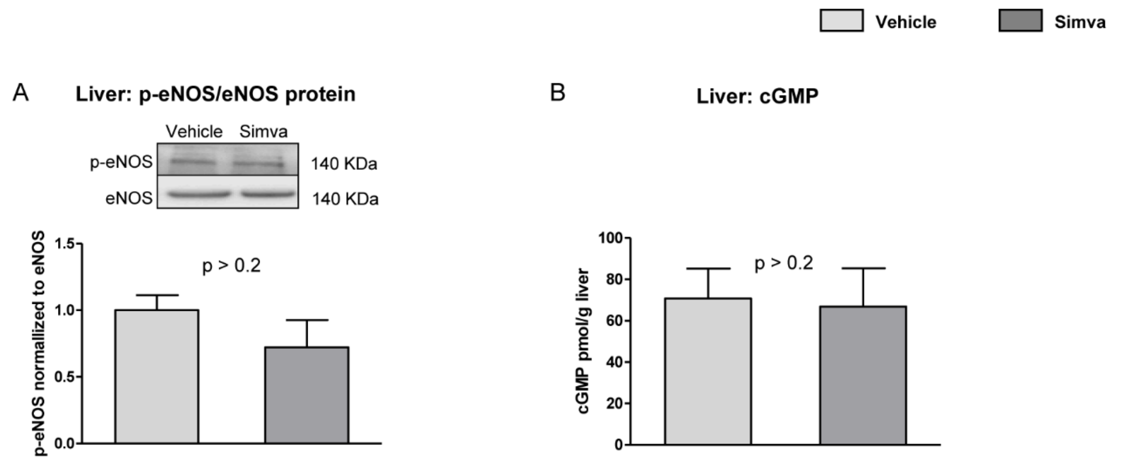

Supplementary Figure 2. NO-pathway in aged rats with aCLD treated with simvastatin or vehicle. (A) Representative blots of eNOS and p-eNOS, normalized to GAPDH in liver tissue from aged rats with aCLD treated with simvastatin or vehicle. **(B)** Hepatic levels of cyclic GMP determined in rats described in A. n=10 (A and B) per group. Results represent mean ± S.E.M.

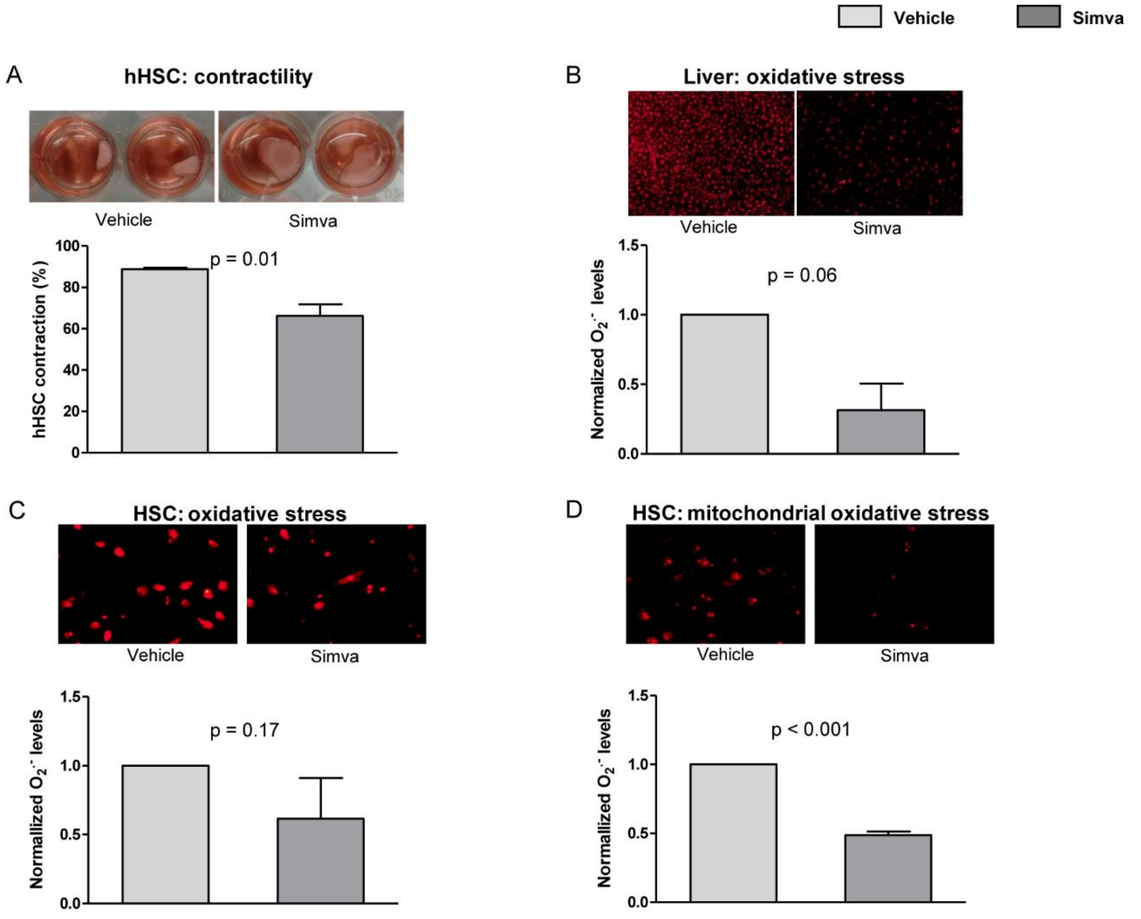

Supplementary Figure 3. Simvastatin promotes HSC de-activation and reduction of oxidative stress. (A) Representative images of HSC contractility and their corresponding quantification in cells freshly isolated from human aged cirrhotic livers treated *in vitro* with simvastatin or vehicle. **(B)** Representative images of $O_2^{\cdot-}$ levels in rat liver tissue (red fluorescence) & corresponding quantification. **(C)** Representative images of $O_2^{\cdot-}$ levels in cirrhotic rat primary HSC (red fluorescence) & corresponding

© 2019. Maeso-Díaz R et al. Published online at http://www.aginganddisease.org/EN/10.14336/AD.2019.0127

quantification. **(D)** Mitochondrial $O_2^{\cdot-}$ levels (red fluorescence) determined in cirrhotic rat primary HSC. n=3 (A, B) and n=5 (C, D) per group. Results represent mean ± S.E.M. All images 400X, scale bar=50μm.

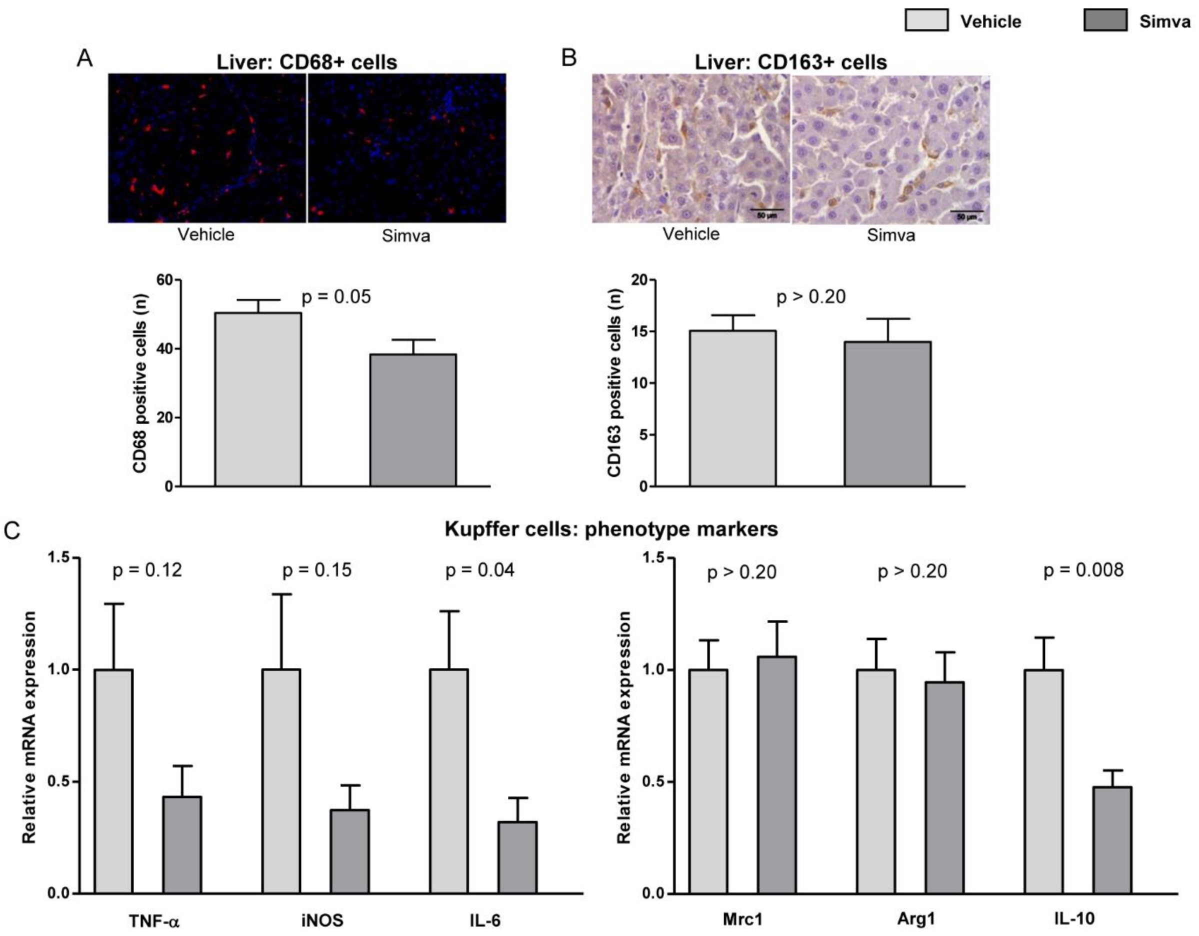

Supplementary Figure 4. Simvastatin enhances macrophages deactivation. (A) Representative images of CD68 immunofluorescence in liver tissue from aged cirrhotic rats treated with simvastatin or vehicle, and corresponding quantification. **(B)** Representative images of CD163 immunohistochemistry in liver tissue from rats described in A and its quantification. **(C)** Expression of TNF-α, iNOS, IL-6, Mrc1, Arg1 and IL-10 in liver tissue from rats described in A. n=10 per group. Results represent mean ± S.E.M. All images 400X, scale bar=50μm.
